# Prognostic molecular markers with no impact on decision-making: the paradox of gliomas based on a prospective study

**DOI:** 10.1038/sj.bjc.6604378

**Published:** 2008-05-27

**Authors:** M Wager, P Menei, J Guilhot, P Levillain, S Michalak, B Bataille, J-L Blanc, F Lapierre, P Rigoard, S Milin, F Duthe, D Bonneau, C-J Larsen, L Karayan-Tapon

**Affiliations:** 1 Neurosurgery Department, University Hospital, Poitiers, France; 2 Neurosurgery Department, University Hospital, Angers, France; 3 Clinical Investigation Centre, INSERM 802, University Hospital, Poitiers, France; 4 Department of Pathology, University Hospital, Poitiers, France; 5 Department of Pathology, University Hospital, Angers, France; 6 Department of Medical Genetics, University Hospital, Angers, France; 7 Laboratory of Molecular Oncology, University Hospital, Poitiers, France

**Keywords:** adult gliomas, outcome prediction, markers, RT-PCR, LOH, decision-making

## Abstract

This study assessed the prognostic value of several markers involved in gliomagenesis, and compared it with that of other clinical and imaging markers already used. Four-hundred and sixteen adult patients with newly diagnosed glioma were included over a 3-year period and tumour suppressor genes, oncogenes, *MGMT* and *hTERT* expressions, losses of heterozygosity, as well as relevant clinical and imaging information were recorded. This prospective study was based on all adult gliomas. Analyses were performed on patient groups selected according to World Health Organization histoprognostic criteria and on the entire cohort. The endpoint was overall survival, estimated by the Kaplan–Meier method. Univariate analysis was followed by multivariate analysis according to a Cox model. p14^ARF^, p16^INK4A^ and *PTEN* expressions, and 10p 10q23, 10q26 and 13q LOH for the entire cohort, *hTERT* expression for high-grade tumours, *EGFR* for glioblastomas, 10q26 LOH for grade III tumours and anaplastic oligodendrogliomas were found to be correlated with overall survival on univariate analysis and age and grade on multivariate analysis only. This study confirms the prognostic value of several markers. However, the scattering of the values explained by tumour heterogeneity prevents their use in individual decision-making.

The estimated incidence of adult gliomas in Europe and the United States is 4–5 per 100 000 inhabitants. The most malignant form is glioblastoma multiforme (GBM), the commonest primary brain tumour in adults with an estimated incidence of 2–3 per 100 000 inhabitants and that has a very poor prognosis with a median survival of about 40–48 weeks despite surgery, radiotherapy and chemotherapy (Laws *et al*, 2003; Stark *et al*, 2005). Chemotherapy provides a sometimes questionable and, at best, limited benefit (Stewart, 2002; Stark *et al*, 2005; Stupp *et al*, 2005). However, the development of new treatment protocols over recent years (Board and Jayson, 2005) and the emergence of new therapeutic concepts (Board and Jayson, 2005; Galanis *et al*, 2005; Haas-Kogan *et al*, 2005; Mellinghoff *et al*, 2005; Hall and Vallera, 2006; Sathornsumetee *et al*, 2007) have accentuated the need to characterize potential candidates for treatments as precisely as possible. Although the diagnostic standard is the World Health Organization (WHO) histoprognostic classification, which can be used to define homogeneous patient groups in terms of prognosis (Kleihues and Webster, 2000), some patients do not present the expected outcome. In parallel, recent progress in molecular biology has allowed determination of numerous markers in routine clinical practice, but raises the question of their capacity to improve patient management (Boudreau *et al*, 2005). The purpose of the present study was to answer this question with respect to certain markers that have already been shown to play a role in gliomagenesis. Published studies on this subject are retrospective and usually based on patients selected according to a particular histoprognostic group or treatment modalities. To the best of our knowledge, this is the first large-scale prospective study based on all adult gliomas.

## PATIENTS AND METHODS

*Study design and inclusion criteria* Two centres participated in the study: the Poitiers and Angers university hospitals, in France. Tissues from these glial tumours were collected during surgery after obtaining signed informed consent from all patients, approval from the Poitou-Charentes ethics committee and in accordance with the precepts established by the Helsinki Declaration. All adult patients in whom a diagnosis of glioma was suspected were prospectively and consecutively preincluded and were definitively included after confirmation of the diagnosis based on WHO histopathologic criteria. These glial tumors encompass: (i) diffusely infiltrating astrocytomas (i.e., diffuse astrocytomas, anaplastic astrocytomas and glioblastoma multiforme), (ii) oligodendroglial tumours (i.e., oligodendroglioma and anaplastic oligodendroglioma) and (iii) mixed gliomas (i.e., oligoastrocytoma and anaplastic oligoastrocytoma) (Kleihues and Webster, 2000). These patients had no particular past medical history, especially no history of brain surgery, brain radiation therapy or chemotherapy. Distribution of the patients according to the WHO classification is presented in Table 1. Tumour diagnosis and grading were established according to the WHO criteria (Kleihues and Webster, 2000) and were systematically revised in each centre by two expert neuropathologists. Four-hundred and sixteen patients were included from 1 September 2002 to 31 December 2005, and patients not satisfying the inclusion criteria after quality control were excluded from the analysis. Median follow-up is 14 months for all patients, and 29 months for patients who were alive. This series also included three tumour-free patients, operated for refractory epilepsy, obtained from Neurobiotec^®^ (Lyon, France) after ethics committee approval. The main demographic data are presented in Table 1. The primary endpoint was overall survival (OS).

### Methods

The following molecular markers were included in this study: (i) expression of oncogenes *EGFR*, *PDGF* and *VEGF*, (ii) expression of tumour suppressor genes p14^ARF^, p16^INK4A^ and *PTEN*, (iii) expression of O_6_-methylguanine DNA methyltransferase (*MGMT*) and (iv) expression of the catalytic subunit (*hTERT*) of telomerase. In addition, cytogenetic parameters, including loss of heterozygosity (LOH), were also collected: 1p, 2p23, 9p, 10p, 10q23, 10q26, 11q, 13q, 17q, 19q, 22q. Clinical data were recorded: age, treatment received, comprising various combinations of surgery, radiotherapy and chemotherapy, including temozolomide (TMZ), OS, histoprognostic types and grades according to WHO grading criteria. Finally, preoperative CT and MRI imaging data systematically performed without and with injection of contrast agent and were recorded. Gene expression was determined by quantitative RT-PCR using the Taqman^®^ gene expression assays kit for each gene, and the device ABIPrism 7000 (Applera^®^) was used. The method used has been previously described (Wager *et al*, 2006). Three modalities of gene expression were defined according to the following cutoff values: decreased: more than one and a half times lower than the control value, normal: from one and a half times lower to one and a half times higher than the control value and increased: more than one and a half times higher than control value. LOH was determined by the microsatellite method. Microsatellite markers and cytogenetic bands and the corresponding genes, when they are known, are shown as additional data. The end point was the correlation between the parameters available on inclusion and OS. Two types of parameters were selected: those already known to be independent prognostic factors and those tested by studying the levels of expression and LOH. This study is not a therapeutic trial. Individual therapeutic options and treatments were proposed and decided independently of inclusion in the study. Of note, TMZ was used since the start of the study (2002). Details of patient treatment appear on Table 2.

### Statistical analysis

Data were collected from the date of inclusion. Differences between groups in presenting characteristics were tested using the Mann–Whitney *U*-test. OS was analysed by calculating the time interval between date of inclusion and date of death from any cause, or date of the last follow-up for surviving patients, and then estimated by the Kaplan–Meier method. The following clinical and biological features were analysed as potential prognostic factors for survival: age, grade (‘high’ *vs* ‘low’), treatment (surgery+radiotherapy+TMZ, *vs* surgery+radiotherapy±other chemotherapy, *vs* others), loss of heterozygosities, relative expression of p14^ARF^, p16^INK4A^, *PTEN*, *EGFR*, *PDGF*, *VEGF*, *MGMT* and expression of *hTERT*. Tested transcripts were categorized in three groups according to the fold change as compared with control, cutoff being set at 1.5 above for overexpression level or 1.5 below the normal expression value for underexpression level. Age and *hTERT* expression were categorized according to the upper and lower quartiles of their respective values. All variables were assessed in univariate analysis using the two-tailed log rank or Wilcoxon test as appropriate. To summarise prognostic information, variables found to be associated at the 20% level with the outcome were entered into a Cox regression model on the basis of likelihood ratio test and potential interactions were tested. A step-down procedure allowed to retain those variables adding to each other prognostic information. Levels of significance were represented by *P*-values derived from two-sided tests. A *P*-value <0.05 or less was considered to indicate statistical significance. SAS v 8 (Statistical Analysis System, Cary, NC, USA) software package was used.

## RESULTS

Expression values are presented in Table 3.

### Univariate analysis

#### Relation of expressions with OS

**Figure 1:**
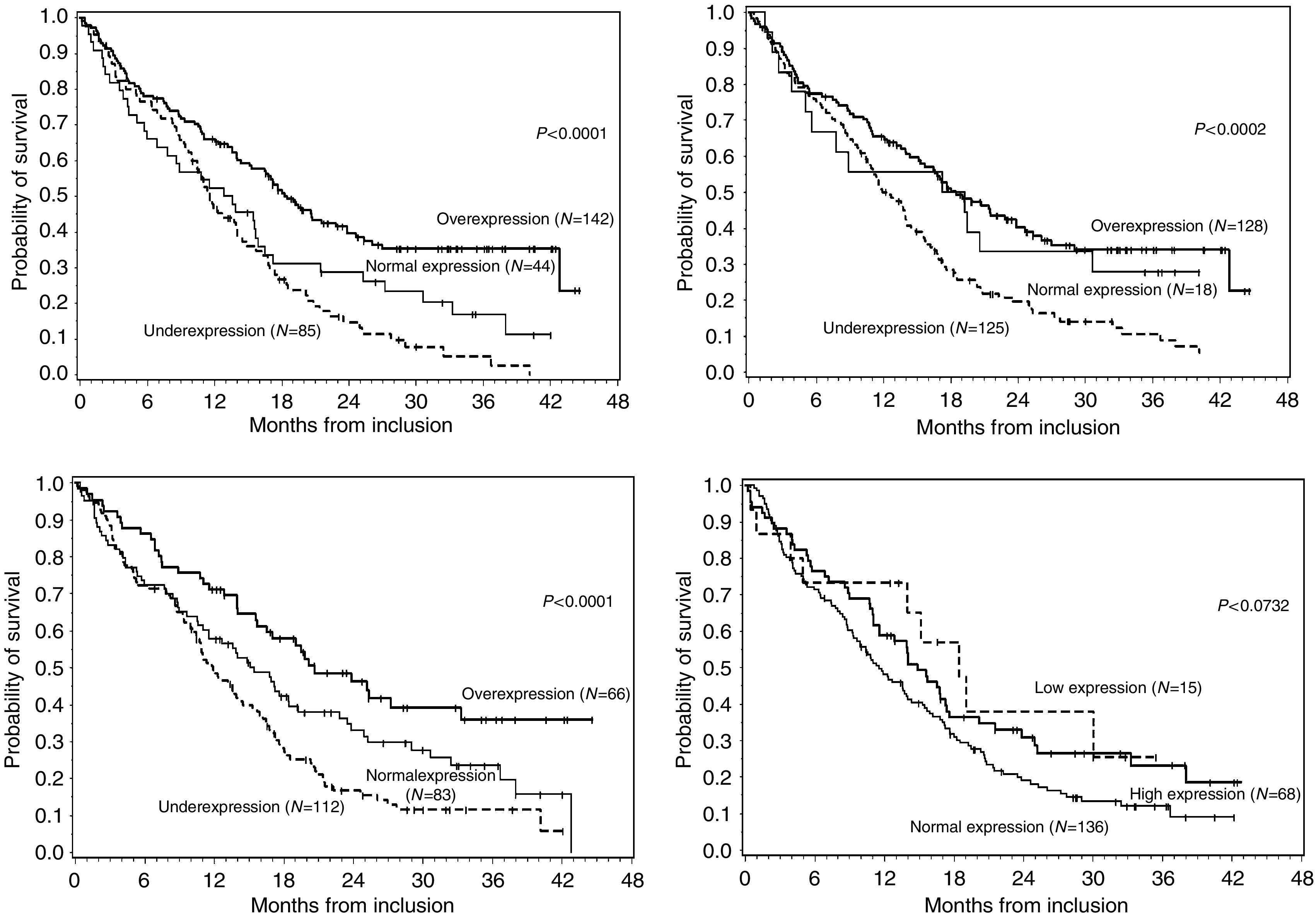
Relationship between p14ARF, p16 INK4A and *PTEN* expressions and Kaplan–Meier estimates of overall survival in the entire series. *hTERT* expression is correlated to overall survival in high-grade tumours, but not in the entire series.

The prognostic importance (Figure 1 and Table 4A) of clinical features at baseline, treatment, relative gene expression of p14^ARF^, p16^INK4A^, *PTEN*, *EGFR*, *PDGF* and *VEGF* and the expression of *hTERT* in univariate analysis is summarized in Table 4A.

Of clinical relevance, three factors were considered: age, grade (‘high’ *vs* ‘low’) and contrast enhancement (*P*<10^−4^). When tested individually, p14^ARF^, p16^INK4A^ and *PTEN* provided prognostic information (*P*<0.05), overexpression being associated to a better survival (Figure 1). Among other factors, the relative expression of *VEGF*, the expression of *hTERT* were considered as potential prognostic factors, underexpression or low expression, respectively, showing a slight advantage, as well as *EGFR* with a very slight advantage when overexpression. Treatment was also considered and categorized as follows: surgery+radiotherapy+TMZ, *vs* surgery+radiotherapy±other chemotherapy *vs* others.

No correlation was observed between *MGMT* expression and survival, either for the entire cohort or for each WHO histopathologic group but a correlation was observed between *MGMT* expression and grade classified as ‘high-grade’ (higher expression) *vs* ‘low-grade’ (*P*=0.0126). No correlation was observed between astrocytic or oligodendroglial tumour phenotypes and the level of *MGMT* expression.

#### Relation of LOH with OS

**Figure 2:**
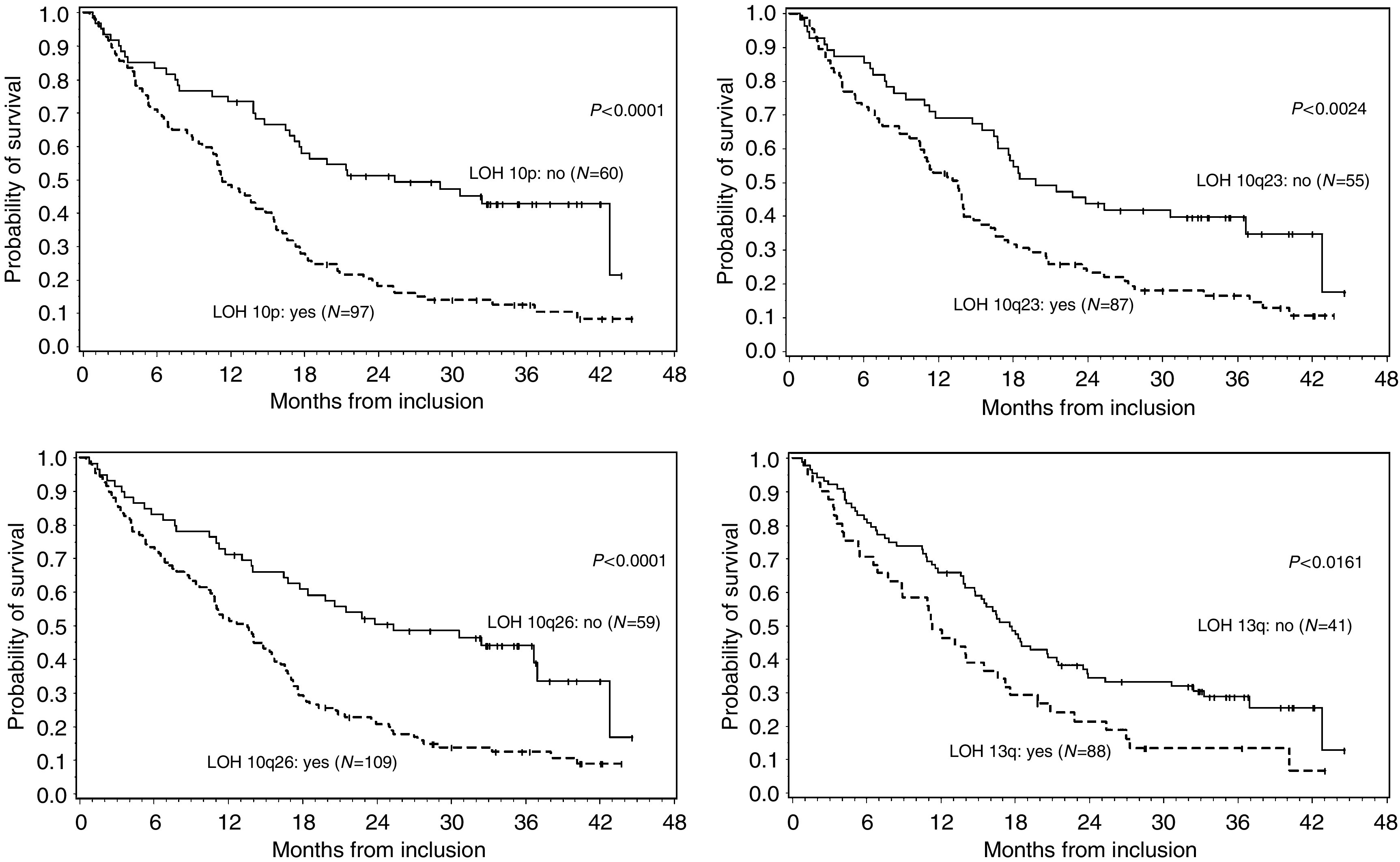
Relationship between 10p15, 10q23, 10q26 and 13q LOH, and Kaplan–Meier estimates of overall survival.

**Figure 3:**
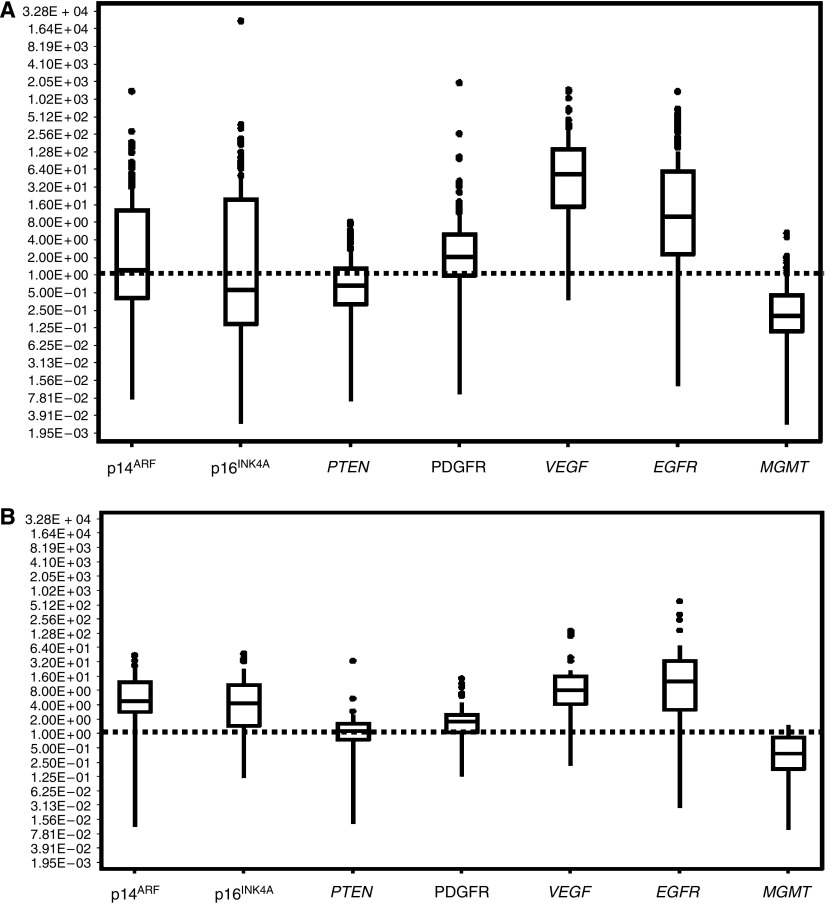
Box-plots showing scattering of gene expression values, for low-grade (**A**) and high-grade (**B**) tumours, respectively.

For the entire cohort, 10p15 (carrying KLF6), 10q23 (*PTEN*), 10q26 (*DMBT*_1_) and 13q (*Rb*_1_) LOH were correlated with OS. 10q26 was correlated with OS of high-grade tumours and also with grade III oligodendrogliomas OS. No correlation was observed between OS of patients with low-grade tumours and the various laboratory parameters studied (Figures 2 and 3 and Table 4A).

### Multivariate analysis

When the eight factors that were identified in univariate analysis (*P*<0.20), treatment, grade and interaction between grade and treatment were entered into a Cox model, only three variables were selected with a *P*-value less than 0.05, namely age (*P*<10^−4^), grade (*P*=0.0017) and *VEGF* (*P*=0.0071) (Table 4B). The relative expression of *VEGF* was not retained after stepwise regression procedure. Factors that were considered adding to each other prognostic information were age and grade, which remained highly significant. The final model is presented in Table 4B.

*MGMT* was of no prognostic value by univariate analysis. However, when this factor was added into the Cox model based on previously published therapeutic approaches considerations, the results remained strictly similar.

## DISCUSSION

The correlations with OS observed in this study confirm data already reported for smaller retrospective series. These results will be discussed globally after a few comments on each of the main markers considered.

### Expressions

A very strong analogy was observed for the results concerning p14^ARF^ and p16^INK4A^, which is coherent with their localization corresponding to the ‘*INK4A locus*’. Hypermethylation of p14^ARF^ has been associated with progression of astrocytomas (Watanabe *et al*, 2007) and has been described as a factor of poor prognosis of grade II diffuse gliomas (Watanabe *et al*, 2003), although its prognostic value in oligodendroglioma is more controversial (Korshunov and Golanov, 2001; Kamiya and Nakazato, 2002). p16^INK4A^ deletion is a factor of poor prognosis of these tumours (Jeon *et al*, 2007). Globally, these data are coherent with concomitant inactivation of these genes during the late stage of gliomagenesis. Loss or inactivation of *PTEN* has been described in solid tumours of various organs, and it is mutant in about 30% of GBM, but not in low-grade gliomas and very rarely in anaplastic gliomas (Duerr *et al*, 1998; Zhou *et al*, 1999). *EGFR* is the main oncogene identified in brain tumours, but its prognostic value is controversial (Chakravarti *et al*, 2001; Muracciole *et al*, 2002; Miracco *et al*, 2003; Shinojima *et al*, 2003; Heimberger *et al* 2005a, 2005b; Kleinschmidt-DeMasters *et al*, 2005; Lopes-Gines *et al*, 2005; Quan *et al*, 2005; Rich *et al*, 2005), although a fairly strong correlation with OS was observed in the present study. *PDGF* is often overexpressed in gliomas, with an elevation correlated with tumour grade (Shih and Holland, 2006) and was considered to be predictive of poor prognosis in a study on grade II tumours (Varela *et al*, 2004). The interest in *PDGF* is largely due to the growing importance, over recent years, of tyrosine kinases as useful targets in the treatment of cancer (Board and Jayson, 2005). The expression of certain *VEGF* receptors appears to be correlated with glioma grade (Plate *et al*, 1992), conferring a diagnostic and prognostic value and also making *VEGF* a candidate for targeted therapies (Grau *et al*, 2006). The *hTERT* gene codes for the catalytic subunit of telomerase and the potential diagnostic value of *hTERT* expression was recently highlighted (Chakravarti *et al*, 2001; Tchirkov *et al*, 2003; La Torre *et al*, 2005; Boldrini *et al*, 2006; Maes *et al*, 2007; Shervington *et al*, 2007).

Methylation of the *MGMT* promoter, a frequent phenomenon, makes gliomas more sensitive to alkylating agents by reducing gene expression (Esteller *et al*, 2000). Several teams have reported this increased chemosensitivity for various types of gliomas (Balana *et al*, 2003; Paz *et al*, 2004; Hegi *et al*, 2005; Watanabe *et al*, 2005; Herrlinger *et al*, 2006; Chinot *et al*, 2007) and *MGMT* status, either methylation or level of expression, has become a predictive marker of chemosensitivity. However, although determination of this marker is recommended in the context of clinical trials involving alkylating agents, it cannot be recommended in routine clinical practice (Stupp and Hegi, 2007). It must be kept in mind that most previously reported correlative studies examined *MGMT* promoter methylation, whereas in the present study, the authors chose to examine MGMT gene expression and that comparison of data obtained from these different approaches are not appropriate. *MGMT* appeared to be more markedly overexpressed in low-grade tumours but *MGMT* expression was not correlated with a specific phenotype, and its level of expression has no specific prognostic value. Patients with low *MGMT* expression treated with alkylating agents presented a better OS than patients not treated by alkylating agents, but it must be remembered that this was not a clinical trial with randomised treatment.

### LOH

Tumour progression reflects genetic instability that can be expressed by several alternative pathways in the same tumour phenotype, resulting in a correlation between the degree of anaplasia of a tumour and the percentage of LOH detected in tumour cells, although a constant profile of loss or gain related to a particular tumour phenotype has not been demonstrated. This phenomenon has been described in meningiomas, but, to the best of our knowledge, has not been previously described on a very large prospective series of adult gliomas. As for meningiomas, no constant expression profile, based on single LOH or combinations of LOH, was found to be correlated with a particular tumour phenotype. However, some LOH appear to be correlated with OS: 10q26, which carries *DMBT*_1_ (deleted in malignant brain tumours), is a possible tumour suppressor gene in glial tumours (Mollenhauer *et al*, 1997). It is generally accepted that passage from an anaplastic phenotype to a GBM phenotype is associated with 10q LOH (Fujisawa *et al*, 1999; Daido *et al*, 2004), although the role specifically played by *DMBT*_1_ in this progression is controversial (Sasaki *et al*, 2002). Taken all together, these results suggest firstly that an increased frequency of this LOH is associated with increasing grade. Secondly, its very strong correlation with survival in the grade III oligodendrogliomas group suggests a participation of *DMBT*_1_ at the anaplastic stage of gliomagenesis. LOH10p is more frequent in high-grade than in low-grade tumours and is also more frequent in tumours with a large oligodendroglial component. The present study demonstrated a link between 10p LOH and poorer survival in the overall cohort but not in the various histoprognostic groups. As 13q LOH was first observed in low-grade gliomas, it was initially thought to be involved at an early stage of gliomagenesis (Lee *et al*, 1995). However, it was subsequently associated with anaplastic astrocytomas and GBM (Wooten *et al*, 1999) and appeared to be mutually exclusive with 19q LOH (Nakamura *et al*, 2000).

### General discussion

All of the correlations observed with OS in this study cohort, probably as a result of the large number of patients, which compensates for the scattering of data, therefore, confirm the results reported in the literature. The correlation between each marker studied and OS can, therefore, be confirmed or excluded for the overall cohort. The results of studies such as this one, can, therefore, confirm the involvement of these markers in gliomagenesis and constitute a valuable tool. However, these results have certain limitations, as these correlations with OS are observed more rarely and become less robust when based on smaller sample sizes and the very broad scattering of the results for these markers prevent any extrapolation of the conclusions drawn from this study cohort to individual patients. These conclusions, therefore, have no practical impact as an aid to individual decision-making. As this scattering is due to tumour heterogeneity, neither studies based on larger series nor studies conducted over longer periods would be able to eliminate this obstacle. Moreover, the relationship between markers and OS was observed at relatively late stages of these gliomas, and no positive correlation was observed for low-grade tumours. Finally, these relationships would only provide a diagnostic contribution when the correlation observed is higher than that of already identified markers. However, the markers correlated with OS in this study were much less robust than other previously identified prognostic indicators, such as age and treatment modalities. These markers, therefore, do not provide crucial contribution in clinical practice at the present time.

## CONCLUSIONS

Application of relevant data provided by molecular biology to the treatment of cancers is a major challenge in medical oncology. In this context, it is a widely held belief that, in the years to come, these data will allow treatment tailored to each individual patient's tumour. However, the results of the present study, which, to our knowledge, is the largest prospective study ever performed to evaluate the prognostic value of biological markers in adult gliomas, tend to suggest that these markers will be of limited value for individual treatment decisions, particularly for the most heterogeneous, that is, high-grade, tumours. If tumour markers were to have a role in treatment decisions in adult gliomas, it would probably be limited to less heterogeneous, that is, low-grade, tumours, as illustrated by the study of certain p73 isoforms. The absence of correlation between LOH and OS of low-grade tumours observed in this study could be due to lower genetic instability, whereas the absence of correlation with gene expressions could be due to the fact that the genes studied here are involved in later stages of gliomagenesis.

*MGMT* has a special place in this context, as, although it has a poor specific prognostic value, it can be used to predict the chemosensitivity of these tumours. Although it is recommended to include *MGMT* in clinical trials of chemotherapy, it cannot be recommended as part of everyday clinical practice. For all of these reasons, it would, therefore, be unrealistic to expect any contribution of these markers to treatment decisions for adult patients with glioma.

When this study was initiated, tumour stem cell and pluripotent stem cell biology was only in the early stages. A growing body of literature has demonstrated the importance of these stem cells to propose an explanatory model for gliomagenesis that could possibly open the way to major therapeutic progress, as these cells constitute the genuine target of treatments. Preclinical animal models reproducing all phases of gliomas could also be used to develop a similar approach to that conducted in this study, based on whole tumours and not stem cells, which can now be specifically isolated and studied. Progress in tumour stem cell (TSC) biology has led to identification of cells possessing the specific characteristics of true glioma stem cells, specific markers (e.g. CD133), capacity for self-renewal and capacity for differentiation into the various cell lines of brain tissue (neuronal, glial) (Gilbertson and Rich, 2007). These results have led to the development of a gliomagenesis model directly resulting in GBM, which postulates the malignant transformation of these cells at the time of ‘insults’, which selectively affect the genes involved in the initial steps of carcinogenesis. Access to these tumour stem cells has now been defined by tumour culture protocols, which will allow molecular studies on much more homogeneous populations. This type of approach applied to individual tumors should result in much more effective use of markers currently identified in heterogeneous cohorts. At the same time, this type of approach would also allow a more accurate definition of the limits of treatments (e.g. resistance of TSC to radiotherapy and acquisition of resistance to chemotherapy) (Rich, 2007).

## Figures and Tables

**Table 1:** Demographic and pathological characteristics (*N*=296)

**Histologic subtypes and grades**	**Patients number *N*=296**	**Patients (%)**	**Age (years) (minimum–maximum)**
Grade II astrocytomas	10	3.38%	45 (20–69)
Grade III astrocytomas	8	2.70%	59 (40–74)
Grade II oligoastrocytomas	10	3.38%	44 (35–63)
Grade III oligoastrocytomas	21	7.09%	53 (22–76)
Grade II oligodendrogliomas	31	10.47%	41 (24–76)
Grade III oligodendrogliomas	40	13.51%	52 (24–79)
Glioblastomas	169	57.09%	64 (30–80)
Giganto-cellular glioblastomas	5	1.69%	56 (45–73)
Undetermined malignant gliomas	2	0.68%	52 (46–59)
Low-grade gliomas	51	17%	
High-grade gliomas	245	83%	

Grades II and III: according to the World Health Organization

**Table 2:** Treatment modalities

	**Grade II tumours *N*=51**	**Grade III tumours *N*=69**	**Grade IV tumours *N*=176**
*(A) General data – grades according to World Health Organization*			
Gross complete removal (%)/biopsy (%)	38 (75%)/13 (25%)	43 (62%)/26 (38%)	124 (70%)/52 (30%)
Radiation therapy Yes (%)/no (%)	10 (20%)/41 (80%)	39 (57%)/30 (43%)	123 (70%)/53 (30%)
Chemotherapy yes (%)/no (%)	20 (39%)/31 (61%)	51 (74%)/18 (26%)	118 (67%)/58 (33%)
			
*(B) Details of combined treatment modalities – grades according to World Health Organization*			
Removal	*N*=38	*N*=43	*N*=124
S only	25 (49%)	5 (7%)	14 (8%)
S+RxTh *n* (/%)	2 (4%)	7 (10%)	23 (13%)
S+RxTh+TMZ *n* (/%)	3 (6%)	11 (16%)	69 (39%)
S+RxTh+ChTh *n* (/%)	0	8 (12%)	10 (6%)
S+TMZ *n* (/%)	2 (4%)	4 (6%)	7 (4%)
S+ChTh *n* (/%)	6 (12%)	8 (12%)	1 (1%)
			
Biopsy	*N*=13	*N*=26	*N*=52
B only *n* (/%)	4 (8%)	6 (9%)	17 (10%)
B+RxTh *n* (/%)	0	0	6 (3%)
B+xTh+TMZ *n* (/%)	3 (6%)	9 (13%)	12 (7%)
B+RxTh+ChTh *n* (/%)	2 (4%)	4 (6%)	3 (2%)
B+TMZ *n* (/%)	1 (2%)	4 (6%)	12 (7%)
B+ChTh *n* (/%)	3 (6%)	3 (4%)	2 (1%)

B, Biopsy; ChTh, chemotherapy other than TMZ; RxTh, radiotherapy; S, surgery; TMZ, temozolomide.

**Table 3:** Expression data for the entire series: histologic subtypes and grades according to the World Health Organization

	***p14***	***p16***	**PDGF**	**EGFR**	**PTEN**	**VEGF**	**MGMT**	**hTERT**
A II	10	10	10	10	8	8	8	10
	**4.91** (*0.01–28.50*)	**2.30** (*0.15–51.27*)	**2.07** (*0.73–9.85*)	**10.20** (*2.64–337*)	**1.14** (*0.65–2.00*)	**4.88** (*1.04–15*)	**0.28** (*0.10–1.37*)	**0.0002** (**0–0.07)**
OA II	10	10	10	10	8	10	8	10
	**4.28** (*1.03–45.57*)	**2.75** (*0.12–49.87*)	**1.88** (*0.57–11.96*)	**29.71** (*0.97–154*)	**1.16** (*0.01–2.57*)	**10.74** (2.51*–*154)	**0.45** (*0.03–1.007*)	**0.001** (*0–0.52*)
OD II	28	28	27	28	26	27	26	28
	**5.91** (*0.62–47.5*)	**5.26** (*0.50–39.40*)	**1.74** (*0.12–14.83*)	**12.91** (*0.03–639*)	**1.20** (*0.34–34*)	**7.01** (*0.21–146*)	**0.40** (*0.01–1.59*)	**0.001** (**0–0.62)**
**Low-grade tumors**	48	48	47	48	42	45	42	48
	**5.06** (*0.01–47.5*)	**4.39** (*0.12–51.27*)	**1.82** (*0.12–14.83*)	**12.91** (*0.03–639*)	**1.17** (*0.01–34*)	**8.00** (*0.21–154*)	**0.40** (*0.01–1.59*)	**0.001** (**0–0.62)**
A III	6	6	6	6	6	6	6	6
	**1.18** (**0.39–19.43)**	**2.43** (*0.05–23821*)	**1.36** (*0.09–42.52*)	**16.11** (*1.72–55.33*)	**1.69** (*0.19–5.78*)	**26.06** (*5.98–744*)	**0.24** (*0.13–1.97*)	**0.005** (*0–0.02*)
OA III	21	21	21	21	21	21	21	21
	**1.78** (*0.22–75.58*)	**0.56** (*0.009–398*)	**2.11** (*0.43–35.51*)	**8.40** (*0.73–302*)	**1.28** (*0.26–3.14*)	**13.00** (*2.13–266*)	**0.20** (*0.01–1.44*)	**0.004** (*0–0.17*)
OD III	35	35	35	35	34	34	34	35
	**3.53** (*0.19–49.42*)	**1.78** (*0.01–195*)	**2.97** (*0.46–13.45*)	**13.83** (*0.92–739*)	**1.07** (*0.09–8.22*)	**42.69** (*1.31–272*)	**0.24** (*0.06–5.39*)	**0.004** (*0–0.17*)
GBM	157	157	157	157	154	154	153	157
	**0.91** (*0.01–1488*)	**0.48** (*0.003–23821*)	**2.07** (*0.01–266*)	**11.24** (*0.01–1478*)	**0.53** (*0.33–1.13*)	**73.77** (*0.38–1562*)	**0.20** (**0.003***–***5.58)**	**0.01** (**0–0.29)**
**High-grade tumors**	224	224	224	224	220	220	219	224
	**1.24** (*0.01–1488*)	**0.58** (*0.003–23821*)	**2.08** (*0.01–1978*)	**10.44** (**0.01***–***1478)**	**0.71** (**0.01***–***8.22)**	**54.95** (*0.38–1562*)	**0.21** (*0.003–5.58*)	**0.01** (**0–0.29)**
**Entire series**	272	272	271	272	262	265	261	272
	**1.73** (*0.01–1488*)	**1.141** (*0.003–23821*)	**1.986** (*0.01–1978*)	**10.85** (*0.01–1478*)	**0.79** (*0.01–34*)	**41.07** (*0.21–1562*)	**0.23** (*0.003–5.58*)	**0.01** (*0–0.62*)

A, astrocytoma; GBM, glioblastoma II, grade II, III, grade III; OA, oligoastrocytoma; OD, oligodendroglioma.

**Table 4:** Relationship between markers and overall survival

**(A) Univariate analysis**				
**Variables**	**All grades *P*-value**			
Age (<48 years /48–68 years/>68 years)	<10^−4^			
Grade II III IV	<10^−4^			
Contrast enhancement	<10^−4^			
Treatment	0.0692			
0: No surgery+radiotherapy				
1: surgery+radiotherapy+chemotherapy other than TMZ				
2: surgery+radiotherapy+TMZ				
				
				
**Expression**	**Grade II**	**Grade III**	**Grade IV**	**All grades**
P14^ARF^^a^	0.2441	0.004	0.9708	<10^−4^
P16^INK4^^a^	0.4188	0.0001	0.9357	0.0002
PTEN^a^	0.1499	0.1478	0.0667	0.0001
hTRT^a^	0.3566	0.0286	0.1846	0.0732
EGFR^a^	0.4303	0.2626	0.0250	0.1969
VEGF^b^	0.9794	NA	0.1007	0.0548
PDGFR^c^	0.3658	0.6677	0.4809	0.2877
MGMT^a^	0.7493	0.2668	0.4896	0.7423
				
**LOH**	**Grade II**	**Grade III**	**Grade IV**	**All grades**
LOH10p (KLF6)	0.2360	0.0493	0.6124	<10^−4^
LOH10q23 (PTEN)	0.0509	0.1805	0.9487	0.0024
LOH10q26 (DMBT_1_)	0.3839	0.0041	0.8795	<10^−4^
LOH13q (Rb_1_)	0.5345	0.7662	0.5278	0.0161
				
**(B) Cox model multivariate analysis**				
	**Final model Cox regression model**			
**Variable**	**Hazard ratio**	**95% hazard ratio confidence limits**		***P*-value**
Age	2.345	1.871	2.940	<0.0001
Grade	2.413	1.869	2.357	<0.0001

LOH, loss of heterozygozity; NA, not applicable; TMZ, temozolomide Grades II, III, IV according to World Health Organization.

a Classified as overexpression, median expression and underexpression

b Classified as overexpression, and other

c Classified as Low expression, normal expression and high expression
